# Supplementary material for: Modeling glioblastoma heterogeneity as a dynamic network of cell states
Source: Mol Syst Biol. 2021 Sep 16;17(9):e10105. doi: 10.15252/msb.202010105 (PMC8444284; doi:10.15252/msb.202010105)
Supplement: Supplementary file 6 — Source Data for Figure 5 [file MSB-17-e10105-s004.zip › Figure5A_sourcedata/GSEA_3017/hallmarks_stateD.GseaPreranked.1621934542601/HALLMARK_INFLAMMATORY_RESPONSE.html]

Details for gene set HALLMARK\_INFLAMMATORY\_RESPONSE[GSEA]

|  || Dataset | state23017 |
| Phenotype | NoPhenotypeAvailable |
| Upregulated in class | na\_neg |
| GeneSet | HALLMARK\_INFLAMMATORY\_RESPONSE |
| Enrichment Score (ES) | -0.3834956 |
| Normalized Enrichment Score (NES) | -1.5124542 |
| Nominal p-value | 0.056712963 |
| FDR q-value | 0.14193712 |
| FWER p-Value | 0.833 |
Table: GSEA Results Summary

  

Fig 1: Enrichment plot: HALLMARK\_INFLAMMATORY\_RESPONSE      
 Profile of the Running ES Score & Positions of GeneSet Members on the Rank Ordered List

  

| PROBE | GENE SYMBOL | GENE\_TITLE | RANK IN GENE LIST | RANK METRIC SCORE | RUNNING ES | CORE ENRICHMENT || 1 | HAS2 |  |  | 4 | 0.596 | 0.0507 | No |
| 2 | MYC |  |  | 77 | 0.313 | 0.0236 | No |
| 3 | AXL |  |  | 242 | -0.259 | -0.0795 | No |
| 4 | EIF2AK2 |  |  | 340 | -0.267 | -0.1301 | No |
| 5 | ATP2A2 |  |  | 433 | -0.277 | -0.1759 | No |
| 6 | LY6E |  |  | 437 | -0.278 | -0.1532 | No |
| 7 | NAMPT |  |  | 450 | -0.279 | -0.1372 | No |
| 8 | ATP2C1 |  |  | 505 | -0.286 | -0.1530 | No |
| 9 | ABCA1 |  |  | 563 | -0.293 | -0.1706 | No |
| 10 | CSF1 |  |  | 614 | -0.303 | -0.1817 | No |
| 11 | BST2 |  |  | 617 | -0.303 | -0.1559 | No |
| 12 | FZD5 |  |  | 661 | -0.312 | -0.1608 | No |
| 13 | ITGB8 |  |  | 694 | -0.318 | -0.1567 | No |
| 14 | PDPN |  |  | 889 | -0.369 | -0.2729 | No |
| 15 | DCBLD2 |  |  | 957 | -0.397 | -0.2887 | No |
| 16 | CHST2 |  |  | 1081 | -0.460 | -0.3420 | Yes |
| 17 | SLC1A2 |  |  | 1107 | -0.471 | -0.3187 | Yes |
| 18 | ATP2B1 |  |  | 1129 | -0.495 | -0.2902 | Yes |
| 19 | SLC4A4 |  |  | 1203 | -0.587 | -0.2934 | Yes |
| 20 | SERPINE1 |  |  | 1218 | -0.617 | -0.2485 | Yes |
| 21 | F3 |  |  | 1241 | -0.673 | -0.2047 | Yes |
| 22 | CDKN1A |  |  | 1306 | -1.046 | -0.1597 | Yes |
| 23 | CCL2 |  |  | 1317 | -1.880 | 0.0023 | Yes |
Table: GSEA details [plain text format]

  

Fig 2: HALLMARK\_INFLAMMATORY\_RESPONSE: Random ES distribution      
 Gene set null distribution of ES for **HALLMARK\_INFLAMMATORY\_RESPONSE**
